# Evaluating health policy capacity: Learning from international and Australian experience

**DOI:** 10.1186/1743-8462-6-3

**Published:** 2009-02-26

**Authors:** Deborah H Gleeson, David G Legge, Deirdre O'Neill

**Affiliations:** 1 School of Public Health, La Trobe University, Bundoora, VIC, Australia; 2 Australia and New Zealand School of Government, Carlton, VIC, Australia

## Abstract

**Background:**

The health sector in Australia faces major challenges that include an ageing population, spiralling health care costs, continuing poor Aboriginal health, and emerging threats to public health. At the same time, the environment for policy-making is becoming increasingly complex. In this context, strong policy capacity – broadly understood as the capacity of government to make "intelligent choices" between policy options – is essential if governments and societies are to address the continuing and emerging problems effectively.

**Results:**

This paper explores the question: "What are the factors that contribute to policy capacity in the health sector?" In the absence of health sector-specific research on this topic, a review of Australian and international public sector policy capacity research was undertaken. Studies from the United Kingdom, Canada, New Zealand and Australia were analysed to identify common themes in the research findings. This paper discusses these policy capacity studies in relation to context, models and methods for policy capacity research, elements of policy capacity and recommendations for building capacity.

**Conclusion:**

Based on this analysis, the paper discusses the organisational and individual factors that are likely to contribute to health policy capacity, highlights the need for further research in the health sector and points to some of the conceptual and methodological issues that need to be taken into consideration in such research.

## Background

### The context for health policy-making

Health policy-makers in Australia face a broad range of entrenched (and in some cases, worsening) public health problems, as well as emerging issues that create challenges for the health system into the future. These include, inter alia: continuing poor Aboriginal health [1,2]; widening inequalities in health [3]; an ageing population [4]; increasing health care costs [5]; environmental destabilisation [6]; rising incidence of chronic disease [7]; and emerging threats to public health, such as new communicable diseases [8]. Social and health problems are also becoming more complex and are increasingly recognised as being inter-dependent [9,10].

For these challenges to be met, health bureaucracies at both Commonwealth and state levels need the capacity to plan effectively and put policies in place to ensure the health system (including health care and public health) is organised, funded, delivered and coordinated in the most effective and sustainable way. Doing so requires overcoming significant barriers arising from the complexity of health system financing and program delivery. For example, rising chronic disease rates mean that the health system must be re-engineered to focus more on prevention. However, the capacity to invest in prevention initiatives is hampered by a health system largely focused on episodes of acute care, fee-for-service funding for general practice, poor coordination between acute and community care sectors, and workforce shortages in most health professions [11]. Other obstacles to health reform include the division of responsibility for health between the Commonwealth and the states (which gives rise to coordination problems, overlap and duplication of services, gaps in service delivery and cost-shifting) [12-14]; the complex mix of public and private insurers and providers [15,16] and the presence of entrenched powerful interests that limit the ability of the state to bring about change [17,18]. Many policy problems (such as social exclusion and health inequalities) also cross jurisdictional and portfolio boundaries and require policy-makers and public sector organisations to work in new ways in order to be effective.

Health policy-makers also face challenges in terms of balancing different health system goals such as equity, efficiency and quality. In an environment of limited resources, there need to be some trade-offs between these goals [19,20]. Ensuring equity through a universal health care system is an important value on which the Australian health system is based [21]; however, balancing universality (i.e. equity with respect to both access and levels of service) with cost containment is a significant issue for policy-makers.

### Increasingly complex policy environments

The policy-making environment is said to have become more complex over the last few decades due to major shifts in the relationship between government and society, brought about by large-scale forces such as globalisation (understood as being the increasingly international nature of the political and economic forces which shape policy) and the increasing use of technology [22-25]. Increased reliance on market mechanisms such as managed competition as policy tools has also resulted in changes to the role of the public service in policy-making. Greater involvement of third parties (including citizens and stakeholders) in service delivery results in more complex policy environments with more players outside government, and creates challenges in terms of involving those who are responsible for service provision in policy development [24,26-28]. These developments shape and reflect an increasingly complex health policy environment.

These changes in the policy environment have resulted in greater uncertainty and complexity in policy-making. It is increasingly difficult to predict the impact of policy changes into the long term. With increasing scale and complexity, balancing the rational/technical and the political aspects of policy development appears increasingly difficult. Emerging ideas in the policy literature such as evidence-based policy-making [29,30] and calls for greater policy coherence or 'horizontal government' [31,32] also bring with them implications for the skills required of policy-makers and for the institutional supports required to change ways of working. Policy-makers increasingly need skills in coordinated and cooperative policy work, networking, negotiating, collaborating, and flexible policy implementation [22,33].

### Policy capacity

From the mid-1990s onwards, there was increasing concern about declining policy capacity in many jurisdictions, and governments in many countries turned their attention to building (or re-building) policy capacity during the period from 1995 to 2005. Concerns about policy capacity have been attributed to a number of shifts in public sector environments, including public management reforms such as privatisation, down-sizing and contracting out as well as shifts in the external environment such as globalisation and changes to the state-society interface [34,35]. Concerns continue to be expressed about the policy capacity of public sector agencies, even in wealthy countries [36]. There is a growing appreciation internationally that capacity building in policy-making can contribute significantly to improved public policy outcomes [23,24,26-28,37] although at this stage there is little empirical evidence to show that this is so.

The term 'policy capacity' is generally used to refer to the capacity of public sector agencies to develop and implement "good" policy (although players outside government, including a range of non-government organisations, universities, research agencies and service organisations also contribute to the policy capacity of nations and states, the term is most often used to describe the policy-making capacity of governments and the public service). However, policy choices are always normative with differential implications for different stakeholders. What constitutes 'good' policy in any particular context may be highly contested [38].

The scholarly literature offers a number of different definitions of policy capacity that highlight different dimensions. Among the more useful of these is the definition offered by Painter and Pierre [1] of policy capacity as "the ability to marshal the necessary resources to make intelligent collective choices about and set strategic directions for the allocation of scarce resources to public ends". At the core of most conceptualisations of policy capacity is the capacity to make decisions or "intelligent choices" [1,35,38,39]. A focus on intelligent choice highlights the quality of public sector policy workers as a central capacity issue, and suggests that recruiting, retaining and developing the "best and brightest" policy workers should be a primary focus of capacity building [39].

Other dimensions of policy capacity suggested by commentators in the political science and public administration literature include the capacity to utilise resources [1,40]; the capacity to implement policy decisions [35,38] and coordination of the policy-making process across government [41]. Peters provides the additional insight that policy capacity includes the ability to make not only incremental choices, but also more strategic choices which involve larger deviations from the status quo [38].

If the capacity of the health sector to develop and deliver effective health policy (however defined) is to be strengthened, a better understanding of the elements of policy capacity is needed. However, empirical research on the topic of health policy capacity is lacking. This paper examines international and Australian experience in conceptualising and evaluating public sector policy capacity more generally and draws out some lessons for building health policy capacity and for further research in this area.

## Methods

This paper reviews publicly available public sector policy capacity research undertaken between 1995–2005 in the United Kingdom, New Zealand, Canada and Australia. A comprehensive literature search was undertaken including the published health policy, public policy and public administration literature (searched using library catalogues, journal and thesis databases) as well as government reports and grey literature (sourced from databases, search engines and government and direct searching of government and organisational websites). The search was restricted to Canada, New Zealand, the United Kingdom and Australia due to their shared political traditions, including the Westminster tradition, and broadly similar approaches to health care delivery. The conclusions of this review ought likewise to be regarded as applying to these kinds of jurisdictions. Evaluations of policy capacity or the quality of policy advice at both federal/national and state/provincial levels were included in the data set. No health sector-specific studies of policy capacity were found, although some studies included health departments among the units surveyed.

Table 1 shows the documents included in the comparative review. The studies were analysed according to the following variables: context and themes; models and methods; findings and recommendations for building policy capacity. In each area, common themes and key differences were drawn out. The analysis was informed by the peer-reviewed literature on policy capacity and public policy as well as the health policy literature. Based on this analysis, some hypotheses about elements of policy capacity in the health sector were developed.

**Table 1:** Documents included in the review

**Jurisdiction**	**Document**
Canada	Canadian Government (1996) Strengthening Our Policy Capacity. Report of the Task Force on Strengthening the Policy Capacity of the Federal Government.
	Manitoba Office of the Provincial Auditor (2001) A Review of the Policy Development Capacity Within Government Departments.

United Kingdom	UK Cabinet Office (1999) Professional Policy Making for the Twenty-First Century. Strategic Policy Making Team.
	Bullock, H, Mountford J et al (1999) Better Policy Making. London, Centre for Management and Policy Studies, Cabinet Office

New Zealand	State Services Commission (1999) Essential Ingredients: Improving the Quality of Policy Advice.
	State Services Commission (1999) Looping the Loop: Evaluating Outcomes and Other Risky Feats.
	State Services Commission (1999) High Fliers: Developing High Performing Policy Units.
	State Services Commission (2000) Pieces of the Puzzle: Machinery of Government and the Quality of Policy Advice.
	State Services Commission (2000) Gaining Through Training: Developing High Performing Policy Advisors.
	Wright (1999) Strategic Policy Advice: Improving the Information Base. New Zealand State Services Commission.
	Wolf (2000) Building Advice: The Craft of the Policy Professional. New Zealand State Services Commission.

Australia	Australian National Audit Office (2002) Developing Policy Advice. Audit Report No. 21, 2001–2002.
	Victorian Auditor-General's Office (2004) Report on Public Sector Agencies: Results of Special Reviews and Other Studies, August 2004.
	Australian Public Service Commission (2004) Connecting Government: Whole of Government Responses to Australia's Priority Challenges. Management Advisory Committee, APSC, Commonwealth of Australia.

## Results

### Introduction to the studies: Context, themes, models and methods

Significant differences existed between the policy capacity studies undertaken in the four jurisdictions, in terms of the context in which they were initiated, the drivers for the review of policy capacity, the actors undertaking the studies, the focus and major themes driving the reviews, the models of policy process underpinning the studies, and the methods used for evaluating policy capacity. These differences are discussed in the section below.

#### Canada

The Canadian Government began to focus on rebuilding policy capacity in the mid-1990s, after two decades of "new public management" and public sector downsizing were perceived to have resulted in a decline of policy capacity [42,43]. A task force instigated by the Clerk of the Privy Council Office in 1995–96 investigated the policy capacity of the federal government and explored ways in which it could be strengthened [44]. Task Force data-gathering processes included interviews with officials in most departments and roundtables with junior policy officers and external policy research experts [44]. Major themes of the ensuing report included strengthening policy capacity across government for dealing with horizontal issues (as well as within departments), workforce development, and building more effective links with the policy research community [44]. The study draws extensively on an analysis of seven "policy functions": theoretical research; statistics, applied research and modelling; environmental scanning, trends analysis and forecasting; policy analysis and advice; consultation and managing relations; communications; and program design, implementation, monitoring and evaluation [44]. This approach aligns closely with rational, "stagist" models of the policy process, such as the Bridgman and Davis [45] policy cycle.

The themes identified in the pivotal Canadian Government Task Force report have been echoed in the scholarly literature from Canada, such as strengthening coordination and coherence across government [31,32], and policy analytic capacity (in particular the challenges of recruitment and retention) [36,39,43]. This report also led to a focus on strengthening policy research capacity, and the Policy Research Initiative was subsequently established to improve the generation and use of research on cross-cutting policy issues [46].

Also in Canada, the Office of the Provincial Auditor in Manitoba Province undertook a smaller scale evaluation of the policy process, organisational structures and processes and policy outputs in 2001. This initiative seems to have been driven by concerns about the inability of briefings and policy documents produced by the bureaucracy to meet the needs of decision-makers; so the emphasis in this report is largely on improving the outputs of the policy process [26]. The methodology involved developing criteria for effective policy development capacity in three domains (policy process, organisational context and the "policy product") based on a literature review and consultations with people involved in policy development. These criteria were used as the basis for interviews with senior managers in departments and political policy-makers including Cabinet Ministers and the Policy Secretariat. Interviewees were asked to rank the criteria according to their relative importance.

#### The United Kingdom

A major program of public sector reform began in the United Kingdom in 1999 with the *Modernising Government *White Paper [27] in which policy-making was identified as one of five key areas for reform. Key themes in the White Paper included "forward looking" and consultative policy-making and "joined-up" government [27]. Improving the use of evidence in policy-making was also a strong focus of *Modernising Government *and the subsequent policy capacity studies undertaken in the UK [[47] p.13]. Academic literature from the UK has also focused extensively on both joined-up government [48,49] and improving the utilisation of research in policy-making [30,47].

The UK Cabinet Office followed *Modernising Government *with a study of "professional policy-making" [37], which involved an audit of policy-making using a model of "modernised policy-making". This study involved collecting case studies of "good practice" policy-making, as well as interviews with officials and advisers, focus groups with policy staff and "recent leavers", and a training needs analysis involving ministers and policy staff [37]. A further study by the Cabinet Office entitled *Better Policy-Making *[50] examined approaches to modernising the policy process, and looked more closely at enabling factors and barriers to change. A survey of civil servants in all ministerial departments was undertaken for this study.

The UK policy capacity initiative was unusual in several respects. First, it was initiated by politicians rather than by public servants – this gave it a breadth and scope beyond the studies done in other countries. It also ensured that the judgements of "good" policy reflected the political priorities and orientation of the government of the day. Second, it was based on a complex conceptual model of policy-making that included a set of characteristics of "modernised" policy-making with three overarching themes and nine core competencies. The UK studies have a strong focus on competencies, many of which (such as "outward looking" and "joined up") seem to be used not only to describe the capabilities of policy staff but to describe organisational capabilities, and, even more broadly, to characterise the approach to "modernised policy-making" [[37] p.12]. The conclusions of this survey, however, appear to be largely embedded in the methods and the assumptions that went into the methods, particularly with respect to the desired policy competencies.

#### New Zealand

In New Zealand, the State Services Commission undertook a suite of studies in 1998–99 as part of a project called "Improving the Quality of Policy Advice". As the title indicates, the project was driven by concerns about the quality of policy advice developed through the bureaucracy [42]. The focus of this project was largely on the quality of inputs (particularly information and research, but also evaluation, consultation and coordination) to the policy process [24,51-56]. Key themes included: lack of effective use of information, evidence and evaluation; emphasis on short-term outputs and "fast solutions" in policy development at the expense of a longer term, more strategic focus; shortages of appropriately skilled staff and lack of policy knowledge and experience [24,51-56]. This project was strongly based in the public policy literature with a number of comprehensive literature reviews [51,52]. Data collection involved interviews with central agency officials and chief executives and policy managers [24]. One of the working papers also examined six high performing policy units, using case studies and interviews [54].

Although the New Zealand studies were quite broad in terms of the issues covered, the emphasis on improving policy advice meant that the recommendations of the studies were generally directed towards senior managers in departments, and were therefore narrower in scope than the some of the capacity building strategies developed in Canada and the UK.

#### Australia

Australian experience with evaluating policy capacity began in the mid-1990s with a series of trials in evaluating the policy advice function of Commonwealth Government central agencies using Policy Management Reviews (PMRs) based on performance assessment techniques [57,58]. PMRs were an attempt to subject the policy development and advice functions of public sector agencies to the same scrutiny as that given to administration at the time, and were driven by concerns with outcomes and accountability [57,58]. These trials raised methodological debates about the criteria for evaluating policy advice (discussed later in this paper). Di Francesco argues that an original intention to evaluate policy advice according to its outputs (such as briefs), the policy itself, and the outcomes (the impact of policy in the real world) was abandoned in favour of a focus on the processes of policy advising [57]. The use of PMRs seems to have petered out after the trials, largely due to a recognition of the limits of applying program evaluation principles to the inherently political policy process [57,59].

At a later stage, performance audits of policy advice systems and processes were undertaken by the Australian National Audit Office [60] and the State of Victoria's Auditor-General's Office [61]. These audits focused largely on improving the process through which policy is made and the quality of policy advice outputs such as briefings and submissions. The scope of these studies is relatively limited in comparison with some of the other studies considered in this paper. The ANAO report was based on discussions with departmental employees and their staff, case studies of quality management arrangements and document analysis [[60] p.13]. Quality management systems for the provision of policy advice were assessed against criteria based on a literature review and other sources and a set of "better practice principles" for enhancing management and quality assurance of the provision of policy advice were developed. The Victorian audit assessed policy briefs and the policy development process against a set of "good practice" principles developed using a literature review and expert interviews [61]. Like the New Zealand studies, these performance audits were focused on the development of policy advice and their recommendations were directed towards the particular departments audited.

The Australian Public Service Commission undertook a major initiative to improve inter-sectoral approaches to policy-making, culminating in the *Connecting Government *report in 2004 [23]. The report was based on a comprehensive literature review and a series of case studies of "whole of government" policy-making. Although the focus of this report is on whole-of-government responses to major cross-portfolio issues, it identifies a number of areas where capacity could be improved and some of the findings relating to institutional culture and the skills set for policy-makers are useful for thinking about departmental capacity. Unfortunately "whole of government" was limited to "'whole of Commonwealth Government", so the challenges of intergovernmental policy coordination were not addressed.

### Key findings and recommendations for building policy capacity

The findings and recommendations of these various policy capacity studies point towards two broad areas as the focus of capacity building: organisational capacity and individual competencies. To analyse the elements of policy capacity in each of these areas, the authors created a table with the main analytical themes, and categorised the issues and recommendations in the documents accordingly, using an iterative process.

The individual competencies include:

• Knowledge and experience

• The practical skills of policy making

• Personal attributes such as creativity, intuition and judgement.

The elements of organisational capacity which emerge from these studies include:

• Access to and use of information and evidence

• Personnel management and workforce development

• Consultation and communication

• Inter-departmental coordination and networking

• Implementation

• Monitoring, evaluation and review

• Strategic management and leadership

• Institutional culture.

The next section of the paper explores findings of the policy capacity evaluations in each jurisdiction in each of these domains.

#### Individual competencies

The knowledge, skills and capabilities of policy staff were frequently referred to in all the studies. In the Manitoba report, the knowledge and skills of policy staff were identified as being the most important factor contributing to excellence in policy development and were also the most frequently cited area needing improvement [[26], pp. 34–36]. Despite this emphasis, however, there was little in-depth exploration of the knowledge and skills that policy practitioners need to do their work and how the presence or absence of these capabilities impacted on the outputs or outcomes of policies.

In this paper the capabilities of policy-makers described in the studies are considered under the headings of: knowledge; practical skills of policy-making; and creativity, intuition and judgement. As the Canadian Government Task Force [44] notes, the skill sets and relevant knowledge requirements differ for different types of policy personnel (with different roles in policy development, implementation and evaluation) and it is the overall mix of skills which is important for policy capacity.

##### Knowledge

Studies identified a number of different types of knowledge important for policy-making. These included knowledge of context, both the context of the problem and of the policy, including the organisational, political and wider social context [[26], p.37,52]. Various disciplines were identified as contributing to policy-making, including law, economics, accountancy, statistics, the social sciences, project management and information technology [37]. Knowledge of systems and developments in other countries and the ability to "learn lessons" from these was also a key competency in developing more "outward looking" policy-making identified by the UK Cabinet Office [37].

##### Practical skills of policy-making

Policy-making skills described in the reports included analytic skills such as the ability to frame problems, appraise research evidence, predict the likely consequences of policy choices and evaluate associated risks [26,37,52]. Skills in the daily work of policy development (such as drafting, researching, consulting, evaluation and project management) were also mentioned [26,37,52]. High level interpersonal and communication skills were highlighted in several studies [23,37,52]. The ability to utilise information technology, to manage risks and to learn new skills were also highlighted by the UK Cabinet Office [37].

##### Creativity, intuition and judgement

These attributes were emphasised in one of the UK reports, which described the need for policy-makers to be "flexible and innovative", willing to question the status quo and prepared to try out new ideas and work in new ways [37]. The Canadian Task Force also discussed the need for intellectual curiosity, intuition and the ability to be "comfortable with the uncertainties of policy-making" [[44], p. 24]. The Manitoba study also highlighted the importance of creativity and good judgement [26]. The emphasis on these attributes in the studies highlights the significance of personal and second-hand experience with cases (stories of episodes of policy-making that illustrate principles and insights that can then be used to inform practice). Cases become an important source of knowledge upon which judgement and intuition are built.

#### Organisational capacity

##### Information and evidence

The importance of adequate and timely information and evidence to inform policy-making was a strong theme across all the studies, and was particularly prominent in New Zealand and the United Kingdom. This reflects a growing emphasis in the published literature on evidence-based (or evidence-informed) policy-making [see, for example, [29,30]]. The studies reviewed were concerned not just with evidence derived from empirical research, however, but also with other types of evidence, including information about the circumstances in which policy is being made (a distinction that is often not clearly made in the evidence-based policy literature).

Concerns about the use of evidence in policy-making were common. The UK Cabinet Office studies found that evidence was not always utilised effectively, even when research had been commissioned by the departments themselves [37]. The Canadian Government study also found that relevant statistics for policy-making were not always available and capacity for applied research and quantitative modelling varied between departments [[44], p. 5]. Models available for forecasting, analysing contingencies and predicting scenarios were not utilised as fully as they could have been [[44], p. 5–6]. The Manitoba Office of the Provincial Auditor [26] also found that there was limited use of both quantitative and qualitative analysis for both issue analysis and for determining policy options.

The New Zealand State Services Commission identified a number of factors affecting the use of information and evidence, including: adequate time to consult with researchers and to commission research where evidence does not exist; skills in using information; ability to coordinate information resources between departments; the production of longer-term and strategic research; and productive relationships with external research organisations [24]. Ideas for improving the use of evidence include commissioning more long-term and strategic research, increasing central-agency expectations and improving coordination across the public service [24].

Many of these ideas in the Canadian and New Zealand studies are echoed in the UK Cabinet Office study *Professional Policy Making for the Twenty First Century*, which identified the need to improve the ability of policy-makers to use evidence and also to ensure that relevant evidence was available [37]. Strategies to achieve this included development of systematic research strategies within the departments, coordination of research effort across government and a range of strategies to improve the accessibility of research evidence including establishing a "Centre for Evidence-Based Policy" to develop international policy networks, databases and information resources. The UK Cabinet Office also advocated the establishment of a "policy researcher" role with a particular focus on gathering evidence and presenting it in a format accessible to policy-makers [37]. Some studies recommended the deliberate development of knowledge management infrastructure to facilitate sharing of knowledge across the bureaucracy. Strategies proposed included policy "knowledge pools" and "knowledge networks". The UK report *Professional Policy Making for the Twenty First Century *[37] recommended the establishment of a policy knowledge pool including information about policies (such as policy objectives, impact assessments and evaluation results, consultation, and evidence drawn upon) in a standard format.

Knowledge management was also highlighted as an important factor in facilitating joined-up responses in the Australian whole-of-government study [23] and identified as a "better practice principle" in the ANAO audit. The ANAO [[60], p. 81] also suggested including directories of subject expertise and information about the policy agenda as well as information about specific projects in the knowledge pools, and recommended cross-agency networks of policy advisers "to identify, research and coordinate policy based on themes".

##### Personnel management and workforce development

A common theme in the studies was the importance of an adequate supply of highly skilled policy personnel and an appropriate mix of skills within units or departments. Staff shortages presented barriers to longer term and more strategic policy-making in many studies. For example, the Canadian Government study [44] noted shortages of key skills, particularly for "policy generalists", and highlighted the importance of personnel management. Areas in which personnel management needed improvement included rotation of staff, recruitment processes, performance review, and mobility and variation in experience. The recommendations for improvements in these areas were mostly cast in terms of self-evident principles; few suggestions were made about how to give effect to these principles.

Training needs assessment and the provision of training for new policy staff, policy staff new to the field and non-policy officers engaged in policy work were recommended by the Canadian Government [[44], p. 24–30]. Access to training in a range of areas (including analytical thinking, particular disciplines important for policy work and the practical skills of policy development, implementation and evaluation) was also identified by senior managers in the Manitoba study as important for improving policy competencies [[26], p. 30]. Lack of both time and money were found to present barriers to training [[26], p. 31].

The UK initiative recommended that training should involve both political and bureaucratic policy-makers (i.e. ministers as well as public servants) [37]; a strategy that encourages relationship building as well as improved knowledge sharing. This was echoed by the ANAO report, which recommended the establishment of a "senior government network in which ministers, senior government officials and other senior policy-makers can meet from time to time for focused seminars on top-level management issues" [[60], p. 108].

##### Consultation and communication

Timely and comprehensive consultation with a range of stakeholders was highlighted as a key factor contributing to good policy outcomes in many studies. Consultation with both internal stakeholders (within the department, other departments, and central government) and external stakeholders (including clients, the public, policy-makers in other jurisdictions, professional associations, academics and researchers) was considered important.

The Manitoba study stressed the importance of involving stakeholders – including clients and the public – in policy development from the beginning, including during the initial data gathering stage [[26], p. 26]. Consultation was one of the core competencies explored by the UK Cabinet Office, which found that although "good practice" communication was reasonably widespread, the resources were not always available to undertake comprehensive consultations [37]. Issues related to consultation were also raised in the Victorian Auditor-General's Office report [61], which identified weaknesses in consultation planning and recommended the use of project management disciplines to promote more systematic planning for consultation.

The ability to keep abreast of international developments and also communicate across jurisdictional boundaries was considered important by the UK Cabinet Office [37], which recommended raising awareness of the "political and wider context", planning for communication, careful targeting, coordination, and fostering relationships with other jurisdictions. Cooperation with "external groups such as community organisations, businesses and other jurisdictions" and "strong external links at the political level – ministers, members of parliament, ministerial staff" were also seen by the Australian Public Service Commission [[23], p. 8] as being important for cross-portfolio policy work. Collaboration with policy researchers was highlighted in the Canadian research, which recommended opportunities for more exchanges [44]. The New Zealand State Services Commission [24] found that building effective consultation into the policy advice process required investment of sufficient time and resources as well as developing particular competencies including negotiation and communication skills. Again, the recommendations for improvements seem to be cast in very general terms and there is little evidence on which to base specific strategies.

##### Inter-departmental coordination and networking

Common findings in the studies included inadequate coordination between departments due to a "silo" mentality and few opportunities for collaborative reflection on best practice. Joined-up approaches across departments (for cross-cutting issues), within departments and between departments, service deliverers and those responsible for implementing policies were a major focus in the UK reports [37]. For these approaches to work, they needed to be supported by compatible information systems, and by organisational cultures and processes [37]. Improving communication between people developing and implementing policies in different portfolio areas was also seen as critical [37]. Sharing best practice was the most frequently identified "enabler" for modernising policy making in the UK report *Better Policy Making *[[50], p. 10]. The Canadian Task Force also recommended the establishment of interdepartmental policy communities among senior policy executives to share ideas about best practice and the struggles of policy work [[44], p. 14].

##### Implementation

Only a few references to implementation were found in the studies reviewed. The lack of attention to implementation in the policy capacity studies is notable, given the extensive literature which points to the importance of full consideration of implementation issues during policy development [45,62-65]. The Canadian study found poor links between policy development and implementation [[44], p. 8]. "Greater consideration to policy implementation" was one of the enablers of change identified in the UK Cabinet Office report *Better Policy Making *[[50], p. 10]. Barriers to the integration of policy development and implementation identified in this report included institutional separation and incompatibility of information technology [[50], p. 41]. There are few clues in the studies, however, as to how links between policy development and implementation could be strengthened. Some strategies that could be explored in further policy capacity research include: improving the capacity for piloting and demonstration; strengthening processes for monitoring and adjustment of incremental policy development and implementation; and involving implementation managers in policy development.

##### Monitoring, evaluation and review

Monitoring and evaluation were areas where weaknesses were commonly identified, as they tended to be neglected, of insufficient quality or did not feed into policy development. For example, the Canadian Task Force found that these functions were frequently separated institutionally from (and therefore poorly integrated with) policy development [[44], p. 11].

Outcome evaluation was identified as a significant input to policy advice by the New Zealand SSC [24], which found that evaluation was most often used for improving delivery and implementation and less frequently contributed to better policy-making. The neglect of evaluation as an input to policy-making was attributed to low demand from ministers, political "short-termism", methodological problems with evaluation, lack of evaluation skills and manipulation of evaluation results for political ends [53]. To improve effective outcome evaluation, the SSC recommended increasing demand from ministers, central agencies and parliament for evaluation information, better specification of outcomes, increasing attention to outcomes (rather than just outputs), improving incentives for reprioritisation and evaluation and improving the skills of policy staff to both carry out evaluations and to manage external evaluations [24].

The Manitoba audit identified deficiencies in performance monitoring of policies and found there was an "ad hoc", rather than systematic, approach to policy evaluation. However senior policy managers did not rank evaluation among the most important criteria for policy capacity and tended to favour a "selective" approach to policy evaluation (as this was perceived to be a more efficient use of resources) [[26], pp. 27–28].

The UK Cabinet Office found there were few opportunities for policy-makers to learn from their own and others' experience [37]. Encouraging a culture of evaluation and improving the quality of evaluations undertaken were both areas that needed improvement [37]. A major recommendation arising from the UK initiative was the use of peer review processes to allow sharing of "good practice" and organisational learning, and also to encourage cultural change [37]. Peer review was also recommended by the ANAO [[60], p. 115] and the Victorian Auditor-General's Office. Other mechanisms suggested by the UK Cabinet Office [37] for improving policy evaluation included strategic management of the evaluation process, better resource allocation, developing a "centre of excellence" devoted to policy evaluation and establishing processes for people implementing policies to feed back information about the effectiveness and acceptability of policies.

##### Strategic management and leadership

Strategic management and leadership of the policy development process were common themes. The Canadian Government Task Force [[44], p. 11] found that systematic management of the policy process was patchy, and the need for it was underestimated. Leadership direction and support was ranked as very important or important by 100% of interviewees in the Manitoba audit of policy capacity [[26], p. 22]. The New Zealand SSC report *High Fliers: Developing High Performing Policy Units *[54] highlighted the importance of leadership and strategic management for improving the performance of "policy units". This included "policy leadership" (in terms of developing a coherent overall direction and policy frameworks) and "management leadership" to provide infrastructure and support to policy work [[54], p. 10]. Recommendations for developing leadership were generally framed around improvements to personnel management (as described above) and institutional culture (see below).

##### Supportive institutional culture

Institutional culture was not a strong (or at least an explicit) focus of many of the studies reviewed for this paper, with the exception of those undertaken by the UK Cabinet Office, the APSC and the New Zealand SSC.

The UK work in particular highlights the importance of institutional culture in recognising the goals of "modernised" policy-making, such as a long-term and more strategic focus [37]. A culture of innovation and preparedness to take risks was considered critical to fostering innovation [37]. A "risk averse" culture was identified as a barrier to improving policy-making [50]. Some recommendations for changing institutional culture included bringing in staff from outside the public service, providing secondments for policy officers and improving networking both within government and with external agencies and other jurisdictions [37].

The APSC's *Connecting Government *report [23] identified a number of features of organisational culture important for the success of whole-of-government approaches. Like the UK reports, it emphasised the importance of innovation and the ability to manage risk. It also highlighted an environment of teamwork and trust and "encouragement of the expression of diverse views" and the ability to "balance the tension between short-term and long-term goals" [23]. Exposure of public servants to different organisational cultures (through secondments and networking) was recommended as one way of fostering a different organisational culture [23].

Clarity of policy direction and policy frameworks was highlighted as important by the Manitoba Office of the Provincial Auditor [[26], pp. 25–6], which described a lack of clear principles or conceptual frameworks for generating or evaluating policy options – although this was identified as a very important aspect of policy development by senior policy managers. It was also found by the New Zealand SSC to be important for improving the performance of policy units [54].

## Discussion

### Implications for policy capacity building and workforce development in the health sector

**Figure 1:**
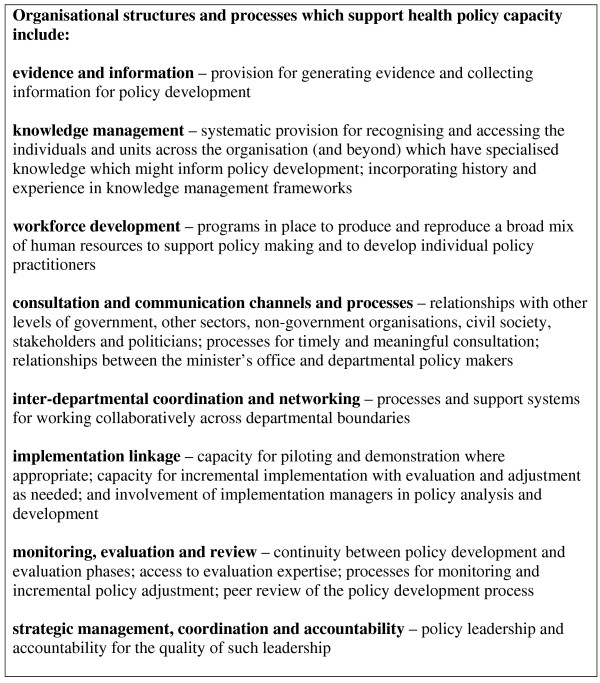
**Organisational structures and processes that support health policy capacity**.

**Figure 2:**
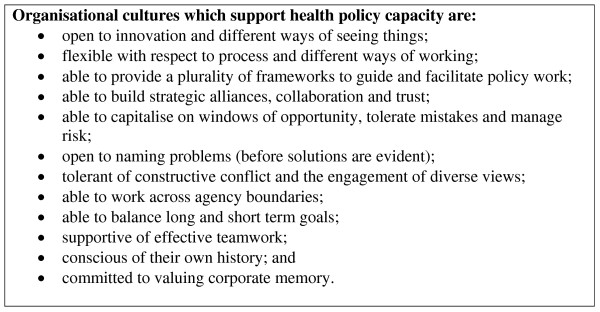
**Features of organisational culture that support health policy capacity**.

**Figure 3:**
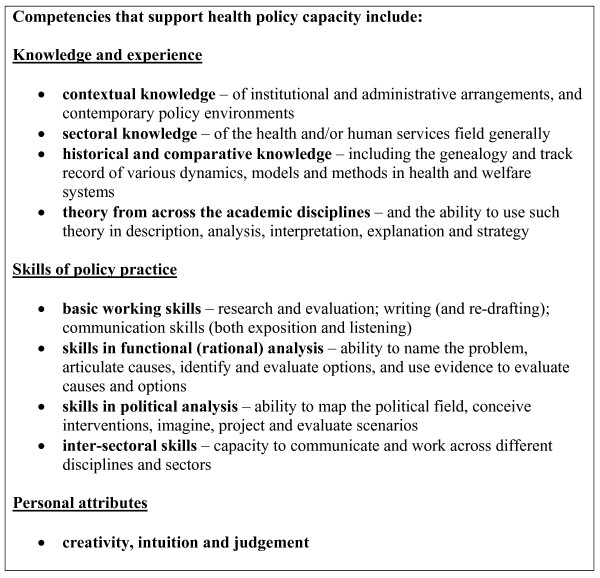
**Individual competencies that support health policy capacity**.

The policy capacity evaluations and audits reviewed in this paper suggest some of the factors likely to contribute to policy capacity in the health sector. Based on the findings of this review, some propositions about the organisational structures and processes, organisational cultures and individual competencies that are likely to contribute to health policy capacity were developed by the authors. These are listed in Figures 1, 2 and 3.

There is clearly a need for further research to explore how these general principles might be applied in the health sector and to examine more closely the institutional arrangements and competencies which create the conditions for good policy outcomes in the health sector. This is the focus of research in which the authors are currently engaged. Questions which might usefully be addressed in such research include:

• To what extent do the generic elements of policy capacity apply to health policy in particular?

• Are some elements of policy capacity more important than others in the context of health policy development and implementation?

Grounding health policy capacity research within the study of the health policy development process would provide the potential to examine more closely the enablers and barriers to good health policy process and enable more specific recommendations for capacity building to be developed.

Jurisdictional differences are clearly important in shaping both policy capacity itself and the sorts of capacity building strategies that might be needed. Some factors that might be important include differences in national systems (such as unitary governments or federal systems), differences in health system contexts, and different institutional arrangements and policy coordination mechanisms (e.g. the Council of Australian Governments). Further research involving different jurisdictions is needed to explore the extent to which comparative differences matter in the context of health policy capacity.

A further issue which should be addressed in future research is the relationship between individual and organisational capacity. Clearly they are closely interrelated. The capacity of individuals is shaped and constrained by the organisational context; the organisational context can only be changed through the efforts of people. Previous policy capacity research does not tell us very much about the nexus between the two, the mechanisms through which they interact and the processes by which change at the individual level can affect the organisation – and vice versa.

### Models and methods for policy capacity research

There are continuing uncertainties about the conceptualisation of policy capacity and the methods for researching it. All the models for conceptualising policy work and the methods for evaluating policy capacity used in the studies reviewed in this paper have their own limitations. Some studies used relatively linear models of the policy process and fairly narrowly conceived methodologies (particularly the state/provincial level audits), which provided little scope for examining the political and contextual aspects of policy-making. Policy work was often conceived as the provision of policy advice to political decision-makers; an approach which may not capture the full range of possible policy engagements and the more active role that policy workers often play in shaping policy.

Most of the studies reviewed for this paper used multiple data collection methods. In most cases, interviews with senior officials were used to collect data. These were often supplemented by interviews with other players such as junior policy officers and external policy research experts in Canada, recent leavers in the UK and cabinet ministers in Manitoba. In some cases interviews were combined with other data collection methods including case studies of policy episodes or policy units, focus group interviews or round tables, surveys, and training needs analysis. A number of appropriate methods are available and the choice of particular methods depends on the context and focus of the study. Triangulation of different methods appears to offer advantages.

Analysis of case studies appears to provide a useful way of exploring the conditions for good policy-making, particularly institutional culture, within context. However, in all the studies reviewed that included analysis of case studies, the cases had been chosen by policy-makers themselves and put forward as examples of good practice. Methods for policy capacity research could be strengthened by more careful selection of case studies according to their potential to illuminate the elements of policy capacity, both positive and negative.

None of the studies included in this review attempted to evaluate policy capacity against the outcomes of the policy process, although some studies evaluated policy capacity in terms of outputs, such as the quality of policy briefs or the satisfaction of ministers.

There has been extensive debate in the literature about the difficulties inherent in evaluating policy outputs and outcomes. First, there are problems attributing policy outcomes to policy capacity or even to particular policies. It is difficult to link policy advice with decisions and their outcomes [66,67]. Even the notion of isolating a discrete area of policy in order to assess outcomes and trace the policy development process is questionable when policies are interwoven and cross-portfolio in nature [66]. Many other variables impact on policy decision-making and its outcomes [57,68], and often the advice of public servants is only one source of advice used by politicians [67]. Second, there is the issue of timeframes. Policy outcomes are often not evident for many years. The policy development itself can often take a long time, and there is often a long lag time between policy development and implementation [67]. Incremental changes to policy during implementation may also mean that the long-term outcomes are no longer traceable to a particular period of policy development [66]. Third, judgements about the value or "goodness" of policy vary widely as there is political and ideological disagreement between different interest groups and actors over goals and outcomes [38,69]. Finally, there are no simple or universally applicable models available for evaluating policy work; it is generally agreed that there would be little value in trying to develop a set of generic criteria to use for evaluating policy capacity [58,69].

For all these reasons, there are no objective standards against which policy outcomes or outputs can be measured. Where policy outputs are evaluated, it is best to employ qualitative methods using professional judgement and peer review [66,67]. Nicholson argued that senior policy advisers who are highly regarded by their peers are likely to be the best judges of the quality of policy advice [67].

Retrospectively judging the outcomes of policy from the position of an outsider carries the additional danger of ignoring the contingencies of all the scenarios that did *not *eventuate, whereas at the point of action, the policy operative would have been confronting a wider range of possible scenarios, all of which needed to be weighed and considered. Any assessment of policy capacity must take into account the range of possible scenarios and contingencies confronting the policy-makers at the time the policy episode took place. This suggests that policy capacity research needs to draw on the accounts of the policy practitioners themselves of their experiences of the policy development process and the environments in which the policy episode took place.

Despite these concerns, some attempts have been made to delineate criteria for evaluating policy capacity. Thissen and Twaalfhoven [69] described three detailed sets of criteria for evaluating policy analytic activities, based on different views of policy analysis: as information provision; as a participative policy-oriented process; and as a set of methods and tools. The framework for organising these criteria distinguishes between inputs, content, process, results, use, and effects. The criteria proposed by Thissen and Twaalfhoven are designed for evaluating policy analytic activities related to specific policy issues, rather than the policy analytic capacity of organisations. Thissen and Twaalfhoven [69] also noted that a single set of universally applicable criteria is not feasible and the choice of criteria depends on the perspective adopted and the particular circumstances of the case. Painter and Pierre [1] suggested a set of evaluative criteria which include a set of values (coherence, "public-regardingness", credibility, decisiveness and resoluteness) and a set of support systems (collective decision processes, planning and evaluation, information and analysis, and coordination procedures). They point out, however, that the evaluative criteria will "necessarily be contested" [1]. While these criteria may provide useful checklists for future policy capacity research, they do not yield "objective" measures of policy process because of the methodological problems noted.

The conceptualisation of policy capacity underpinning much of the policy capacity research has been criticised more broadly for its reliance on a rational model of policy-making that privileges some aspects of policy-making over others. The implicit assumption is that improving and systematising the technical aspects of policy-making will result in better policy. Brans and Vancoppenolle [42] pointed out that this approach conflicts with other perspectives on improving policy-making, such as "interactive governance", which emphasise citizen involvement and participation and the need for government to be responsive and flexible. This suggests there is a dimension to policy capacity that is not amenable to technical solutions and that requires more attention to the interface between the bureaucracy and other players outside government, including industry stakeholders and the wider society [[27], p. 12, 70]. These relational aspects of policy capacity should be a stronger focus in future policy capacity research.

## Conclusion

In the absence of research into health policy capacity, some lessons can be drawn from experience in evaluating public sector policy capacity in Australia and other countries.

The review of public policy capacity research undertaken for this paper suggests a number of elements or domains of policy capacity that could be used to provide a conceptual framework for future research. These include: information and evidence; personnel management and workforce development; consultation and communication; implementation; monitoring, evaluation and review; strategic management and leadership; and supportive organisational culture. Individual competencies for policy practitioners identified in the studies included: knowledge (of context, different disciplines, and systems and developments in other countries); practical skills of policy-making, such as analytic, technical and communication skills; and personal attributes such as creativity, intuition and judgment.

The review of models and methods suggests that future policy capacity research should be grounded in careful description and analysis of the policy process that is not limited to a single narrow perspective. Such research should also use a range of qualitative methods that employ the judgement of policy practitioners themselves in evaluating policy capacity, rather than attempting to measure policy capacity according to "objective" measures of policy outcomes or explicit evaluative criteria.

Health policy is difficult; policy-makers are working with complexity, conflict and uncertainty. However, much is at stake. Further research into the conditions for effective policy-making in the health sector would appear to offer significant returns.

## Competing interests

The authors declare that they have no competing interests.

## Authors' contributions

DHG carried out the data collection and comparative analysis and drafted the manuscript. DGL and DON participated in the design of the study and the data analysis, and critically reviewed drafts of the manuscript. All authors read and approved the final manuscript.
